# Cohort Comparison of Radiographic Correction and Complications Between Minimal Invasive and Open Lapidus Procedures for Hallux Valgus

**DOI:** 10.1177/10711007221112088

**Published:** 2022-07-26

**Authors:** Diogo Vieira Cardoso, Andrea Veljkovic, Kevin Wing, Murray Penner, Oliver Gagne, Alastair Younger

**Affiliations:** 1 Division of Orthopaedics and Trauma Surgery, Geneva University Hospitals, Geneva, Switzerland; 2 Division of Orthopaedics and Trauma Surgery, British Columbia University, Vancouver, Canada

**Keywords:** hallux valgus, Lapidus, first tarsometatarsal fusion, minimally invasive surgery, arthroscopy

## Abstract

**Background:**

The Lapidus procedure corrects hallux valgus first ray deformity. First tarsometatarsal (TMT) fusion in patients with hallux valgus deformity using minimally invasive surgery (MIS) is a new technique, but comparative outcomes between MIS and open techniques have not been reported. This study compares the early radiographic results and complications of the MIS with the open procedure in a single-surgeon practice.

**Methods:**

47 MIS patients were compared with 44 open patients. Radiographic measures compared preoperatively and postoperatively were the intermetatarsal angle (IMA), hallux valgus angle (HVA), foot width (FW), distal metatarsal articular angle (DMAA), sesamoid station (SS), metatarsus adductus angle (MAA), first metatarsal to second metatarsal length, and elevation of the first metatarsal. Early complications were recorded, as well as repeat surgeries.

**Results:**

The mean follow-up was 82 (range, 31-182) months for the open group and 29 (range, 14-47) months for the MIS group. In both techniques, postoperative measures (IMA, HVA, DMAA, FW, and sesamoid station) were significantly improved from preoperative measures. When comparing postoperative measures between both groups, the IMA was significantly lower in the open group (4.8 ± 3.6 degrees vs 6.4 ± 3.2 degrees, *P* < .05). Differential between pre- and postoperative measures for both techniques were compared, and the open group was associated with more correction than the MIS group for IMA (12.4 ± 5.3 degrees vs 9.4 ± 4.4 degrees, *P* = .004) and HVA (25.5 ± 8.3 degrees vs 20 ± 9.9 degrees, *P* = .005). Wound complication and nonunion rates trended higher in the open group (4 vs 0) (*P* = .051).

**Conclusion:**

Both techniques resulted in good to excellent correction. However, the open technique was associated with lower postoperative IMA values and more correction power for IMA and HVA, than the MIS.

**Level of Evidence:** Level III, retrospective cohort study.

## Introduction

Multiple surgical techniques have been described to address hallux valgus deformity.

Among them, the fusion of the first tarsometatarsal joint (TMT) or Lapidus procedure can correct the deformity in 3 dimensions, allowing derotation of the pronated first metatarsal as well as correction of the hallux valgus angle (HVA) and plantar flexion of the first ray.^13^ The original Lapidus procedure as described by Paul W. Lapidus in 1934 has suffered several modifications over the time, mostly regarding the hardware and fixation technique.^21^ Regardless, the Lapidus procedure allows to address severe deformities while offering a stable and reliable fusion.^25^ Because the TMT joint is fused, the risk of recurrence is lower, especially in patients with first TMT joint hypermobility.^3,17^ However, first TMT fusion has been associated with delayed healing, higher malunion, and nonunion rates.^13,20^ Difficulties to reach the more plantar and lateral aspects of the TMT joint with the tendency to remove excessive bone at the medial and dorsal part of the joint in some techniques may contribute to the high rate of first ray shortening and nonunion.^20^ To improve outcomes and decrease complication rates, less invasive techniques have been described.^15,22^ In 2005, Lui et al^15^ described the arthroscopic first TMT fusion technique and concluded that shortening, dorsiflexion, and adduction of the first ray could be minimized with this technique, as the arthroscopic procedure provides more accurate joint preparation, which allows maintaining the subchondral bone intact without taking excessive bone edges. In addition, the arthroscopic technique provides the benefits of minimally invasive surgery, which includes minimal soft tissue damage, reduced postoperative pain, increased bone blood supply, prevention of wound healing complications, and a better cosmetic result.^15,16,22^ In 2020, Vernois and Redfern^22^ described a percutaneous technique for Lapidus arthrodesis using a burr. In their conclusion, they pointed out that this technique is a powerful tool for forefoot deformity corrections, but excessive first ray shortening is a major concern.

Although MIS first TMT fusion techniques have been described, there is almost no information about the clinical and radiologic outcomes of this type of surgery. More important, no comparative studies between open and MIS first TMT fusion have been performed. To our knowledge, only 1 case series (n = 5 patients) has reported the outcomes of the arthroscopic first TMT fusion for hallux valgus correction.^16^

This study aims to assess early radiographic results and complications of the MIS arthroscopic assisted with screw fixation first TMT fusion and compare it with the open procedure in patients with hallux valgus deformity.

## Methods

### Patients

The local ethics committee approved this study. Consecutive patients 18 years of age or older who underwent MIS or open first TMT fusion surgical procedure to treat moderate to severe hallux valgus deformities were reviewed radiographically and screened for complications. As the MIS first TMT fusion was introduced in our institution by the senior author in later 2017, procedures done before were mainly performed through open techniques. Therefore, patients undergoing open first TMT fusion between January 2015 and July 2017 were compared with patients undergoing MIS first TMT fusion between January 2018 and December 2019. The period between July 2017 and December 2017 was considered as learning curve for the MIS technique. Therefore, patients undergoing MIS first TMT fusion during this period were not enrolled.

Exclusion criteria included a simultaneous fusion of the second and third ray, incomplete radiographs, first TMT fusion done for non–hallux valgus procedures, Charcot arthropathy, and fusions of the navicular-cuneiform joint performed at the same time. Data on patient’s baseline characteristics, including age, sex, comorbidities (diabetes), and lifestyle factors (body mass index and smoking status), were obtained from the anesthesia records. Body mass index was calculated by dividing patients’ weight (in kilograms) by their height (in meters squared) according to the World Health Organization.^26^

### Surgical Technique

All the patients were operated on by the senior author (A.Y.).

The MIS technique has evolved in time. Prior to the introduction of Shannon burrs in our country in 2017, the MIS technique was based on the technique described by Lui et al.^15^ After 2017 and before the study, the technique was evolved. The technique involves a percutaneous release of the adductor tendon at its insertion on the plantar aspect of the phalange using a Beaver 64 blade.^5^ A lateral release is performed using the same blade to release the lateral capsule and the lateral sesamoid to metatarsal head ligament under fluoroscopic control. A dorsal medial incision is made on the medial side of the first metatarsal head. To avoid dorsal medial cutaneous nerve injury, subcutaneous dissection is performed using the “nick and spread” technique. A 2 × 12 shannon burr (Stryker, Kalamazoo, MI) is used to cut into the metatarsal head and create a medial eminence resection next to the sagittal grove.

**Figure 1.**
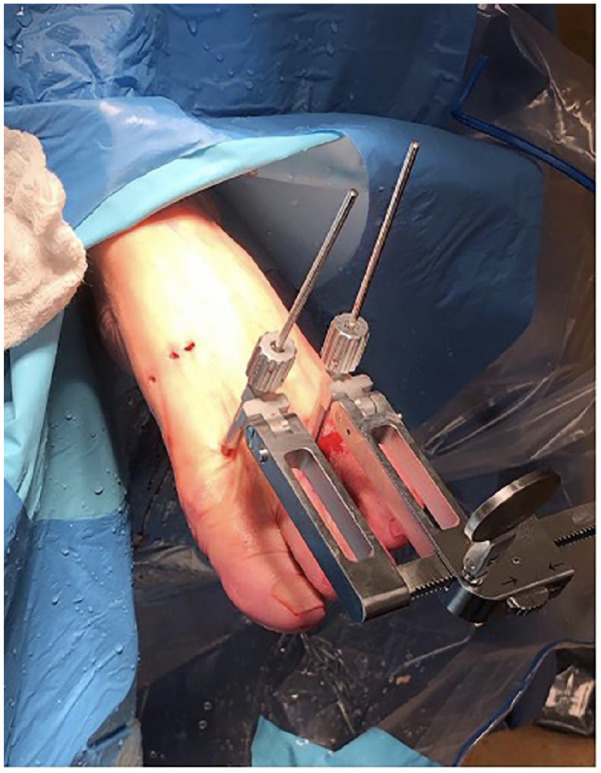
Arthroscopic portal placement and example of a compressor distractor device with 2.4-mm pins in the first and second metatarsal head holding correction.

**Figure 2.**
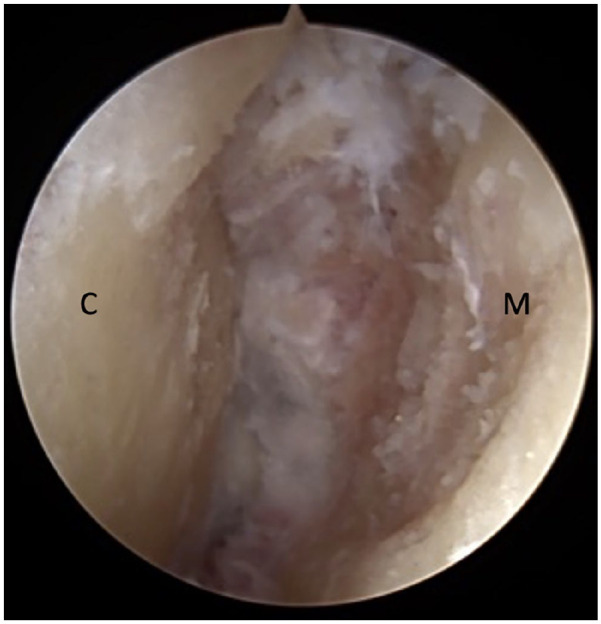
Arthroscopic view of first tarsometatarsal joint after cartilage removal. C, cuneiform; M, metatarsal.

**Figure 3.**
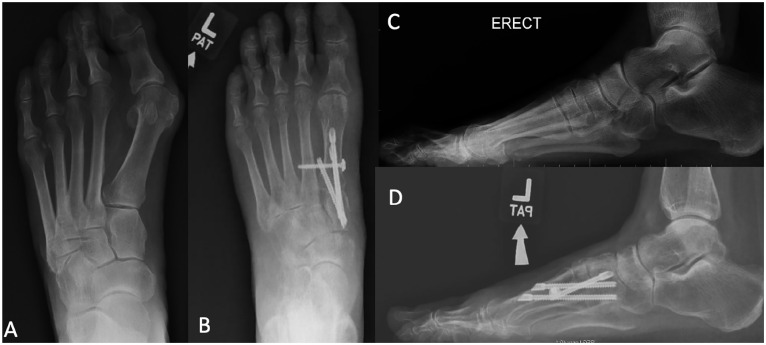
Left foot weightbearing radiographs of a patient undergoing minimally invasive tarsometatarsal fusion surgery. (A) Preoperative AP view. (B) Postoperative AP view. (C) Preoperative lateral view. (D) Postoperative lateral view. AP, anteroposterior.

**Figure 4.**
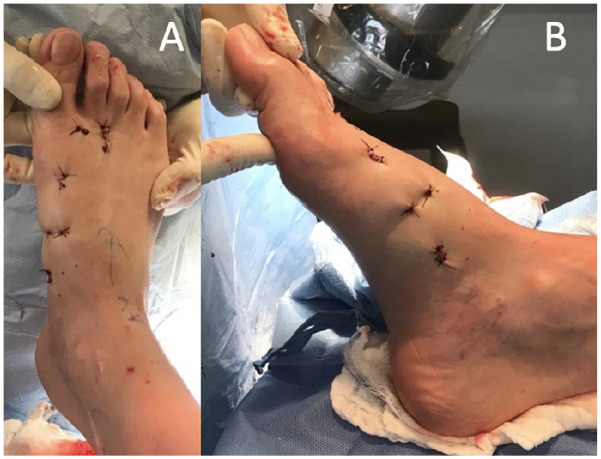
Postoperative clinical aspect of skin incisions with the minimally invasive technique.

The first TMT joint is localized with a Beaver blade and confirmed with fluoroscopy. Two portals are used, one medial and one superomedial (Figure 1). To avoid the tibialis anterior tendon insertion, the medial portal is located at the midpoint between the dorsal and plantar aspect of the TMT joint. A stab skin incision is performed, and the subcutaneous tissue is spread down using a nick and spread technique. The 2 × 12 shannon burr is introduced into the joint and the cartilage sequentially removed using C-arm control and palpation. Alternatively, the 3.1-mm wedge burr can be used for cartilage removal in less tight joints. When the burr is correctly positioned, the 2 bones each side of the joint surface can be felt moving as the burr turns (speed range from 3000 to 18 000 rpm, and aimed for under 6000 rpm).The 2.9-mm, 30-degree arthroscope is introduced, and final debridement and debris removed using a 3.5-mm shaver (Figure 2). As required, an additional superolateral portal can be done to improve joint visualisation and facilitate debridement. Once the joint is fully prepared, the first metatarsal adduction, pronation, and positioning in the sagittal plane are manually corrected. Correction is maintained using a compressor distractor device with 2.4-mm pins in the first and second metatarsal head (Figure 1). A partial-thread, cannulated 3.5-mm intermetatarsal screw is inserted between the first and second metatarsals to hold the position and compress the first to second ray, improving the IM angle. The fixation is then completed with 2 to 3 percutaneous full-thread, cannulated 4.0-mm screws placed crossing and transfixing the TMT joint. An example of pre- and postoperative radiographs showing screw placement is illustrated in Figure 3. Postoperative clinical aspect of skin incisions with the MIS technique are shown in Figure 4. Dressings are used to control the correction.

**Figure 5.**
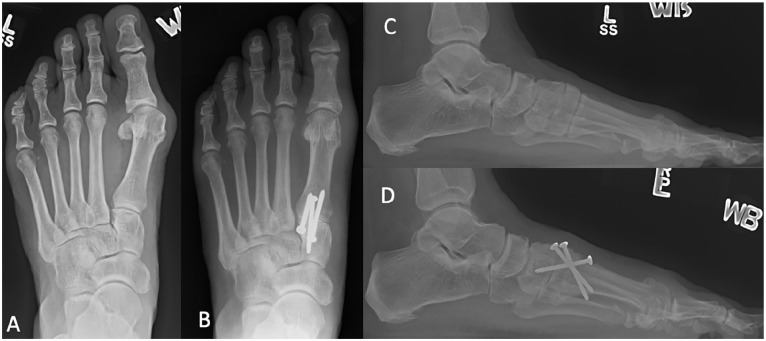
Left foot weightbearing radiographs of a patient undergoing open tarsometatarsal fusion surgery. (A) Preoperative AP view. (B) Postoperative AP view. (C) Preoperative lateral view. (D) Postoperative lateral view. AP, anteroposterior.

The open first TMT fusion was performed according to a previously published technique.^20^ In the open technique, a dorsal incision was used, and 3 screws were placed across the joint. No first to second metatarsal compression screw was used. A medial incision was made and the medial eminence removed with a ronguer. The lateral release was performed open using the distal end of the distal incision. The lateral capsule was released, and the lateral sesamoid released laterally from the adductor and the lateral capsule. The correction was held in 3 planes and a compression distal-to-proximal screw placed. The final 2 screws were placed proximal to distal on the medial cuneiform (Figure 5). In contrast to the MIS technique, the open technique did not include the intermetatarsal screw. Once the first TMT fusion procedure (open or MIS) was concluded, an Akin osteotomy was added if the HVA or the appearance of the first ray showed residual deformity.

### Postoperative Protocol

At the conclusion of the surgical procedure, all patients were placed in a postoperative rigid walker boot. Patients were kept in heel weightbearing and instructed to elevate their foot for the first 2 weeks postoperatively. At the initial 2-week postoperative follow-up, stitches were removed, and patients were allowed full weightbearing as tolerated in a rigid walker boot. Patients were instructed to remove the boot during daily range of motion exercises and weekly physical therapy sessions. Toe spacer or toe alignment splint were not prescribed. At the 6-week postoperative follow-up, the walker boot was discontinued, and patients transitioned to regular comfortable shoes.

### Radiographic Measures

Weightbearing anteroposterior and lateral foot radiographs were assessed preoperatively and at 3, 6, and 12 months postoperatively. Further follow-up was performed if necessary. The following radiographic measures were performed: (1) intermetatarsal angle (IMA)^4^; (2) HVA^23^; (3) metatarsus adductus angle (MAA) according to the Engel angle^6^; (4) distal metatarsal articular angle (DMAA)^12^; (5) foot width (in millimeters)^1^; and (6) sesamoid station (in millimeters): distance between the lateral cortex of the first metatarsal and lateral cortex of the lateral sesamoid (Negative values were considered when the lateral cortex of the first metatarsal was more lateral than the sesamoid. This is modified from the original description, which is a grading of station by Hardy and Clapham.^10^); (7) length of the first metatarsal: difference in length between the first and second rays of the foot (Negative values were attributed to a shorter first metatarsal bone.^9^); and (8) elevation of the first ray: difference in declination between first and second metatarsal measured on the lateral x-ray. (Negative values were attributed when the first ray was plantar to the second ray.^11^)

### Complications and Reoperations

Incidence of postoperative complications was assessed using the patient’s chart up until the time of review and postoperative radiographs and was divided as follows: (1) wound healing problems including dehiscence and wound infection; (2) sensory nerve impairment defined by persisting numbness or paresthesia involving the hallux or surrounding surgical site; (3) fusion nonunion defined by a painful absence of fusion after 12 months postoperatively and requiring revision surgery; and (4) recurrence of deformity defined by symptomatic hallux valgus deformity requiring revision surgery. Furthermore, complications were categorized as minor or major if additional surgery was required or not, respectively. Postoperative hardware-related pain and additional surgery performed for hardware removal was also noted.

### Statistical Analysis

Continuous variables were reported using mean and standard deviation assuming a normal distribution. For continuous variables, the preoperative and postoperative mean were compared using parametric tests and nonparametric tests accordingly. Categorical variables were reported using ratios and percentages. Chi-square tests and/or Fisher exact test were used to compare differences in categorical variables. Statistical analysis was conducted using SPSS. A *P* value of <.05 was considered statistically significant.

## Results

Over the study period, 91 patients had a first TMT fusion for hallux valgus deformity. Forty-seven patients undergoing MIS first TMT fusion were compared with 44 patients undergoing open first TMT fusion.

Baseline demographics of all patients and according to the technique performed are illustrated in Table 1. No significant differences between both groups were observed for comorbidities and lifestyle factors. Preoperative IMA and HVA were slightly higher in the open group (17.2 ± 4.9 degrees vs 15.8 ± 4.6 degrees, and 37.4 ± 6.2 degrees vs 34.4 ± 9 degrees, respectively) (Table 2). Overall, postoperative measures (IMA, HVA, DMAA, FW, and sesamoid station) significantly improved from preoperation. The changes between preoperative and postoperative measures for MAA, length, and elevation of the first ray were not significant.

**Table 1.** Patients Baseline Characteristics (All and by Technique Performed).

	All	MIS	Open
Patients, n	91	47	44
Female, n (%)	81 (89)	43 (91)	38 (86)
Age, y, mean ± SD	60 ± 13	58 ± 12.5	62 ± 13.2
Right foot, n (%)	42 (46)	23 (49)	19 (43)
Lifestyle factors			
BMI, mean ± SD	27 ± 8.7	28.4 ± 11.2	25.1 ± 4.3
Smoking status, n (%)			
Current smoker	14 (15)	8 (17)	6 (14)
Comorbidities			
Diabetes (%)	1 (1)	0	1 (1)

Abbreviations: BMI, body mass index; MIS, minimally invasive surgery.

**Table 2.** Preoperative Radiographic Measures.

	MIS, Mean ± SD	Open, Mean ± SD	*P* Value
IMA (degrees)	15.8 ± 4.6	17.2 ± 4.9	.141
HVA (degrees)	34.4 ± 9.0	37.4 ± 6.3	.091
Foot width (mm)	93.3 ± 6.9	93.1 ± 7.4	.881
DMAA (degrees)	20.4 ± 12.7	24.0 ± 10.5	.131
Sesamoid station (mm)	11.8 ± 2.7	11.3 ± 2.4	.354
MAA (degrees)	21.7 ± 6.8	20.7 ± 6.9	.489
First ray length (mm)	–3.5 ± 4.2	–3.6 ± 3.1	.920
First ray elevation (degrees)	0.1 ± 1.8	0.8 ± 1.6	.139

Abbreviations: DMAA, distal metatarsal articular angle; HVA, hallux valgus angle; IMA, intermetatarsal angle; MAA, metatarsal adductus angle; MIS, minimally invasive surgery.

When comparing postoperative measures between both groups, the IMA was significantly lower in the open group (4.8 ± 3.6 degrees vs 6.4 ± 3.2 degrees) (Table 3). Correction power of both techniques was compared, and the open group showed more powerful correction than the MIS group for IMA (12.4 ± 5.3 degrees vs 9.4 ± 4.4 degrees, *P* = .004) and HVA (25.5 ± 8.3 degrees vs 20 ± 9.9 degrees, *P* = .005). Correction power of both techniques are represented in Table 4. The number of Akin osteotomies was similar between both groups (11 in the open group and 7 in the MIS).

**Table 3.** Postoperative Radiographic Measures.

	MIS, Mean ± SD	Open, Mean ± SD	*P* Value
IMA (degrees)	6.4 ± 3.2	4.8 ± 3.6	.034*
HVA (degrees)	14.5 ± 8.0	11.9 ± 6.2	.084
Foot width (mm)	83.3 ± 6.1	81.1 ± 6.6	.103
DMAA (degrees)	10.3 ± 7.7	11.0 ± 5.3	.187
Sesamoid station (mm)	5.7 ± 4.0	4.9 ± 2.9	.267
MAA (degrees)	20.4 ± 5.7	19.7 ± 5.0	.539
First ray length (mm)	–4.0 ± 3.9	–3.8 ± 2.2	.83
First ray elevation (degrees)	0.5 ± 3.2	1.4 ± 3.0	.597

Abbreviations: DMAA, distal metatarsal articular angle; HVA, hallux valgus angle; IMA, intermetatarsal angle; MAA, metatarsal adductus angle; MIS, minimally invasive surgery; *, significant *P* values.

**Table 4.** Correction Power or Differential Between Pre- and Postoperative Radiographic Measures for Both Techniques.

	MIS, Mean ± SD	Open, Mean ± SD	*P* Value
IMA (degrees)	9.4 ±4.4	12.4 ±5.3	.004*
HVA (degrees)	20.0 ±9.9	25.5 ±8.3	.005*
Foot width (mm)	10.1 ±7.1	12.0 ±6.1	.158
DMAA (degrees)	10.1 ±13.4	13.1 ±10.0	.144
Sesamoid station (mm)	6.1 ±3.9	6.4 ±3.6	.676
MAA (degrees)	1.2 ±6.3	0.9 ±5.5	.814
First ray length (mm)	–0.5 ±4.6	–0.2 ±2.9	.746
First ray elevation (degrees)	–0.4 ±3.5	–0.6 ±2.7	.993

Abbreviations: DMAA, distal metatarsal articular angle; HVA, hallux valgus angle; IMA, intermetatarsal angle; MAA, metatarsal adductus angle; MIS, minimally invasive surgery; *, significant *P* values.

### Complications and Reoperations Rates

Overall, the mean follow-up was 54.6 (range, 14-182) months, 82 (range, 31-182) months for the open group, and 29 (range, 14-47) months for the MIS group. Postoperative complications were observed in 17 (19%) patients. Of these, 12 (70%) were in the open group (*P* = .042). There was a trend toward statistical significancy in wound complications and nonunion rates in the open group (4 wound healing problems and 4 nonunion in the open group vs 0 in the MIS group) (*P* = .051). No differences were observed for neurovascular complications (3 in the MIS vs 1 in the open group, *P* = .617) and for deformity recurrence (2 in the MIS vs 3 in the open group, *P* = .617).

**Figure 6.**
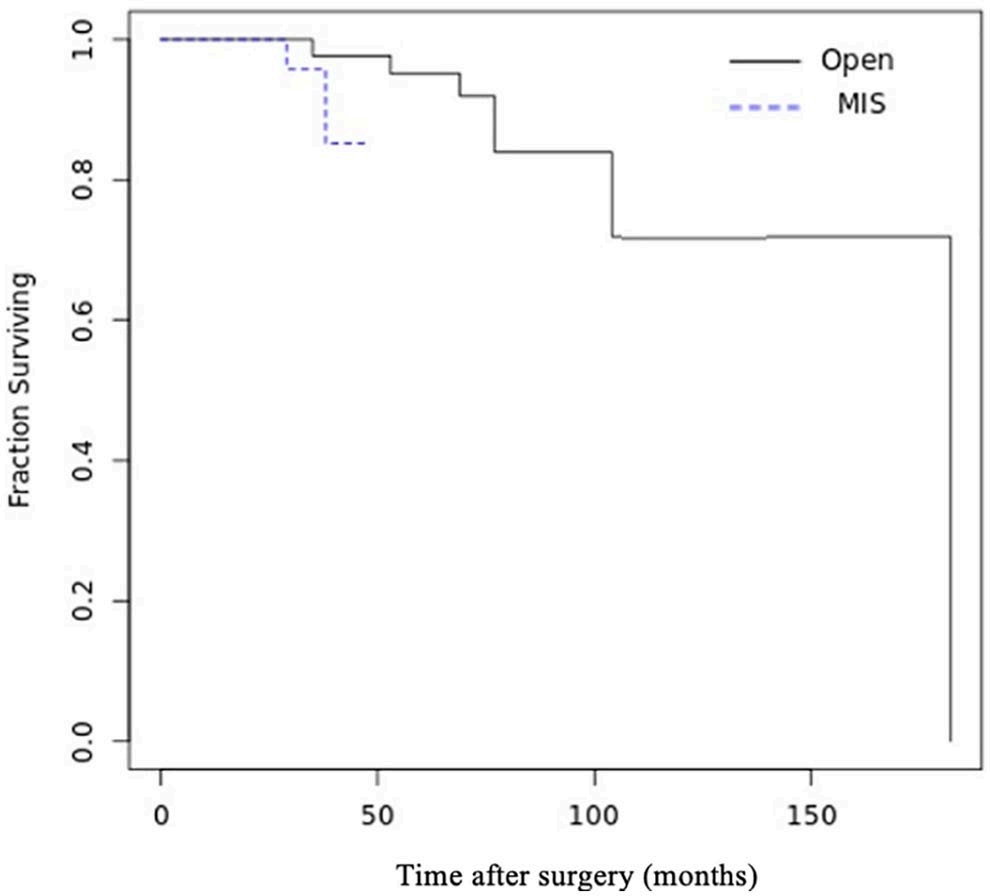
Kaplan-Meier survivorship analysis for major complications requiring revision surgery.

There were 7 major complications in the open group and 2 in the MIS group. In the open group, 4 patients required additional surgery for nonunion, and 3 patients for deformity recurrence. In the MIS group, 2 patients needed revision for deformity recurrence. Survivorship analysis showed increased early repeat surgery in the MIS group at mean 33.5 ± 6.4 months vs 85 ± 47.7 months (*P* = .017), primarily hardware associated (Figure 6).

Hardware-related pain was observed in 17 (19%) patients (9 in the open group and 8 in the MIS group). Of these, 9 (10%) patients underwent surgery for hardware removal (3 in the open group and 6 in the MIS group, *P* = .487).

## Discussion

The percutaneous first TMT fusion performed in patients with hallux valgus deformity is a new technique and, to our knowledge, this is the first article reporting comparative outcomes between MIS and open first TMT fusion. Overall, our results show a significant improvement in the radiologic measures from the preoperative, suggesting that both techniques provide good to excellent deformity correction. However, there was a trend toward less robust IMA and HVA correction with the MIS technique.

Although postoperative IMA absolute values were significantly lower in the open group, it was inferior to 9 degrees in both techniques, which is generally considered a normal IMA.^4^ When comparing the correction power of both procedures, the open group showed significantly more correction of the IMA and HVA than the MIS group (12.4 ± 5.3 degrees vs 9.4 ± 4.4 degrees, and 25.5 ± 8.3 degrees vs 20 ± 9.9 degrees, respectively). The correction power of first TMT fusion through open procedures has been previously reported between 6 and 9 degrees for the IMA, and between 10 and 22 degrees for the HVA.^7,19,20^ In one prospective study involving 46 patients who underwent open first TMT fusion, the mean preoperative IMA improved from 13.5 to 5.7 degrees, with a difference vs baseline of 7.8 degrees, and the HVA improved from 33.8 to 13.9 degrees, with a difference vs baseline of 19.9 degrees.^7^ In comparison to these results, our open group showed higher IMA and HVA correction, which may be explained as being due to higher preoperative deformity in our cohort.

Regarding the MIS results, there are few reports to date, and radiologic outcomes have been reported one single time in one case series involving 5 patients. In this study, published by Michels et al,^16^ the IMA improved from 17.8 degrees preoperatively to 7.2 degrees postoperatively, with a differential of 10.6 degrees. The HVA improved from 42.6 degrees preoperatively to 17 degrees postoperatively, with a differential of 25.6 degrees. In our MIS group, there was a trend toward less correction of IMA and HVA. However, the difference between Michels et al^16^ and our cohort results may be explained by the additional surgical procedure performed in the study of Michels et al. In their cohort, a distal chevron metatarsal osteotomy was performed for all patients in addition to the arthroscopic first TMT fusion. In our cohort, no further metatarsal osteotomy was done in association with the first TMT fusion, and Akin osteotomies were rarely performed. Nevertheless, in retrospective, we think that Akin osteotomies may have been appropriate in some cases to improve final HVA in the MIS group.

Excessive first metatarsal shortening after first TMT fusion has been a major concern, as is a risk factor for developing postoperative transfer metatarsalgia.^8,20^ Using less invasive first TMT fusion techniques with more careful joint preparation would, theoretically, avoid excessive first ray shortening.^15^ The average first metatarsal shortening has been reported between 2.9 and 8 mm with open techniques and 2.7 mm with MIS techniques.^14,16,20^ In our cohort, the differential between pre- and postoperative first metatarsal length was lower than previously reported values, and no significant differences in the postoperative first metatarsal length were observed between open and MIS techniques. We believe that a carefully first TMT joint preparation performed exclusively by hand, and changes in radiograph projections due to slight differences in foot and beamer positions, may have contributed to the lower values of postoperative metatarsal length in our cohort.

Nonunion is one of the most frequent major complication after open first TMT fusion, and its incidence has been reported from 2% to 10%.^18,20,24^ Our results showed a trend toward statistical significance in nonunion rates in the open group as there were 4 nonunion cases in the open group and none in the MIS group. Interestingly, Michels et al^16^ also reported zero cases of nonunion in first TMT fusion with MIS techniques. Similar results were observed for wound complications, with a trend to increased rates in the open group. Although further research is needed to validate this trend, our results suggest that the less invasive first TMT fusion may be associated with lower incidence of complications such as nonunion and wound problems.

Although the open group presented higher complications rates, the survivorship analysis of repeat surgery showed an increased early repeat surgery in the MIS group, and this difference was significant. The learning curve for less invasive procedures is known to be longer and more demanding than that for open surgery.^2^ The senior author progressively introduced the arthroscopic technique in late 2017, and although we have excluded patients treated in the early phase of the learning curve, the longer learning process may have contributed to the increased early revision rates observed in the MIS group. In addition to the learning curve, the MIS technique may be associated with other limitations. The surgical correction of the deformity can be harder to achieve, and placement of screws in the corrected position may be more challenging. We believe that these factors might have influenced the increased early repeat surgery rates observed in our cohort, which were largely hardware removal related.

This study has several limitations, primarily those inherent to all retrospective studies. First, the follow-up period for the MIS group was shorter than the open group, which may be a source of bias when assessing the incidence of complication rates. Studies with longer-term outcomes are warranted to support our findings. Second, as we did not assess clinical outcomes, the clinical relevance of the differences observed in the radiographic measures between both groups is not clear. Further randomized controlled trials with patient-reported outcomes comparing both techniques are required, as this might not be pertinent for patients’ outcomes. Third, the learning curve for the MIS group may have influenced the results observed, and this group needs to be evaluated to determine if the experience with the procedure improves outcomes. As the learning curve and technique are evolving, improvement of correction and reduction of nerve symptoms from the percutaneous technique may be seen in time.

In conclusion, this is the first study comparing radiologic results and complication rates between open and MIS first TMT fusion. Our results showed that both techniques provide significant improvement in radiograph measures from the preoperative with good to excellent deformity correction. Nevertheless, the open technique was associated with lower postoperative IMA absolute values and more correction power for the IMA and HVA than the MIS. The open technique was associated with higher rates of nonunion and wound complications.

## Supplemental Material

sj-pdf-1-fai-10.1177_10711007221112088 – Supplemental material for Cohort Comparison of Radiographic Correction and Complications Between Minimal Invasive and Open Lapidus Procedures for Hallux Valgus Click here for additional data file. Supplemental material, sj-pdf-1-fai-10.1177_10711007221112088 for Cohort Comparison of Radiographic Correction and Complications Between Minimal Invasive and Open Lapidus Procedures for Hallux Valgus by Diogo Vieira Cardoso, Andrea Veljkovic, Kevin Wing, Murray Penner, Oliver Gagne and Alastair Younger in Foot & Ankle International
